# How much variation in oocyte yield after controlled ovarian stimulation can be explained? A multilevel modelling study

**DOI:** 10.1093/hropen/hox018

**Published:** 2017-11-13

**Authors:** Oybek Rustamov, Jack Wilkinson, Antonio La Marca, Cheryl Fitzgerald, Stephen A Roberts

**Affiliations:** 1 Department of Reproductive Medicine, St Mary’s Hospital, Central Manchester University Hospital NHS Foundation Trust, Manchester Academic Health Science Centre (MAHSC), Manchester, England M13 0JH, UK; 2 Primary IVF, Primary Health Care Limited, Brisbane, QLD 4075, Australia; 3 Centre for Biostatistics, School of Health Sciences, Faculty of Biology, Medicine and Health, Manchester Academic Health Science Centre (MAHSC), University of Manchester, Manchester M13 9PL, UK; 4 Research and Development, Salford Royal NHS Foundation Trust, Salford, England M6 8HD, UK; 5 Mother–Infant Department, University of Modena and Reggio Emilia, Modena, Italy

**Keywords:** controlled ovarian stimulation, oocyte yield, ART, IVF, ovarian response, personalized medicine, stratified medicine, multilevel modelling, variation

## Abstract

**STUDY QUESTION:**

How much variation in oocyte yield after controlled ovarian stimulation (COS) can be accounted for by known patient and treatment characteristics?

**SUMMARY ANSWER:**

There is substantial variation in the COS responses of similar women and in repeated COS episodes undertaken by the same woman, which cannot be accounted for at present.

**WHAT IS ALREADY KNOWN:**

The goal of individualized COS is to safely collect enough oocytes to maximize the chance of success in an ART cycle. Personalization of treatment rests on the ability to reduce variation in response through modifiable factors.

**STUDY DESIGN, SIZE, DURATION:**

Multilevel modelling of a routine ART database covering the period 1 October 2008–8 August 2012 was employed to estimate the amount of variation in COS response and the extent to which this could be explained by immutable patient characteristics and by manipulable treatment variables. A total of 1851 treatment cycles undertaken by 1430 patients were included. The study was not subject to attrition, as cancelled cycles were included in the analysis.

**PARTICIPANTS/MATERIALS, SETTING, METHODS:**

Women aged 21–43 years undergoing ovarian stimulation for IVF (possibly with ICSI) using their own eggs at a tertiary care centre.

**MAIN RESULTS AND THE ROLE OF CHANCE:**

Substantial unexplained variation in COS response (oocyte yield): was observed (3.4-fold (95% CI: 3.12 to 3.61)). Only a relatively small amount of this variation (around 19%) can be explained by modifiable factors. A significant, previously undescribed predictor of response was the practitioner performing oocyte retrieval, with 1.5-fold variation between surgeons with the highest and lowest yields.

**LIMITATIONS REASONS FOR CAUTION:**

Although a large number of covariables were adjusted for in the analysis, including those that were used for dosing and determination of the stimulation regimen, this study is subject to confounding due to unmeasured variables and measurement error.

**WIDER IMPLICATIONS OF THE FINDINGS:**

The present study suggests that there are limits to the extent that COS response can be predicted on the basis of known factors, or controlled by manipulation of treatment factors. Moreover, modifiable variation in response appears to be partially attributable to differences between surgeons performing oocyte retrieval. Consequently, consistent prevention of ineffective or unsafe responses to COS is not likely to be possible at present. Our results highlight the importance of blinding surgeons in RCTs. The data also suggest that there is likely to be limited scope for personalized treatment unless additional predictors of ovarian response can be identified.

**STUDY FUNDING/COMPETING INTERESTS:**

J.W. is funded by a Doctoral Research Fellowship from the National Institute for Health Research (DRF-2014-07-050) supervised by S.A.R. The views expressed in this publication are those of the authors and not necessarily those of the NHS, the National Institute for Health Research or the Department of Health. J.W. is a statistical editor of the Cochrane Gynaecology and Fertility Group. S.A.R. is a statistical editor for Human Reproduction. J.W. also declares that publishing peer-reviewed articles benefits his career. A.L.M. has received consultation fees from MSD, Merck Serono, Ferring, TEVA, Roche, Beckman Coulter.

## Introduction

The goal of controlled ovarian stimulation (COS) in ART is to safely obtain enough oocytes to maximize the chance of success in the treatment cycle. Frequently, this goal proves elusive; it has been estimated that 17% of ART stimulation cycles in the UK (Sunkara *et al.*, 2011) and 28% in the USA (Steward *et al.*, 2014) result in the collection of over 15 oocytes, representing increased risk to both the woman (Steward *et al.*, 2014) and any potential offspring (Sunkara *et al.*, 2015). In total, around 12% of IVF cycles in the UK are cancelled owing to poor or excessive ovarian response (Kurinczuk, 2010). If this situation is to be improved, methods to predict and prevent ineffective or unsafe COS responses are required (La Marca *et al.*, 2012, La Marca and Sunkara, 2014). To this end, the predictive value of two ovarian reserve tests (ORT), anti-Mullerian hormone (AMH) and antral follicle count (AFC), has been demonstrated in relation to COS response (Broer *et al.*, 2011, 2013). In addition, the dose-responsiveness of COS response to FSH has also been established (Arce *et al.*, 2014), although this is likely to be limited to patients with sufficient ovarian reserve to permit tailoring (Klinkert *et al.*, 2005, Lekamge *et al.*, 2008). The value of ovarian reserve testing for improving clinical outcomes of ART is less clear; however, with a recent review of RCTs of individualized versus standard doses of FSH noting that only one trial in good prognosis patients had demonstrated an effect on pregnancy (van Tilborg *et al.*, 2016). The same review concluded that tailoring the dose of FSH on the basis of ORTs may improve safety, however. Some support for this is provided by a recent RCT where a multivariable dose selection algorithm increased the proportion of participants obtaining an optimal number of oocytes, albeit using a definition that was not prespecified (Allegra *et al.*, 2017). A second RCT suggested that dose-selection using AMH may reduce the overall proportion of low or excessive responses, although these analyses excluded cycles cancelled for poor response (which occurred more frequently in the personalized group) (Nyboe Andersen *et al.*, 2017).

**WHAT DOES THIS MEAN FOR PATIENTS?**

This paper looks at why women’s bodies respond differently to ovarian stimulation during fertility treatment. The researchers also looked at how women’s responses change if they are treated more than once. The aim of stimulating the ovaries with drugs during fertility treatment is to produce a good supply of eggs to maximize the chances of IVF working. Tests known as ovarian reserve tests (such as AMH and antral follicle count) are often carried out before stimulation begins to see how many eggs the ovaries are likely to produce. This is so that the levels of drugs given can be individually adjusted to try to make sure the woman does not produce too few or too many eggs. The ideal treatment would result in all women producing a similar number of eggs, somewhere in between these two extremes.

The researchers looked at women having treatment at one treatment centre who were given different drug regimes depending on the results of their ovarian test results. They found that differences in the number of eggs obtained were only partially explained by routinely measured characteristics, such as age and ovarian reserve test results. In addition, differences in how women were treated only explained a very small amount.

Because these factors do not explain why women respond so differently, it is not possible to reliably predict how a woman will respond to ovarian stimulation. If two women of similar age with similar ovarian test results are given the same treatment, their responses could be very different. The first woman could get nine eggs, for example, and the second could get as few as four or as many as 19. If the first woman had a second try, she might produce between 5 and 17 eggs. The researchers also found that the doctor carrying out the egg collection also makes a difference to how many eggs are collected.

The paper concludes that personalizing ovarian stimulation to the individual woman can currently only be done in a limited way as we still do not know exactly why women respond differently.

From a statistical perspective, we contend that the challenge of optimizing COS should be viewed as the need to reduce variation in response. This is somewhat different to the typical situation we face when designing and testing interventions, where effectiveness is defined as a shift in an outcome in one direction. In this regard, an understanding of the sources of variation contributing to the distribution of COS outcomes would be advantageous (Senn, 2016). In particular, the amount of unexplained variation represents a limit on our ability to predict response under a given treatment regimen, and the degree to which we can manipulate this response depends on the amount of variation attributable to modifiable factors. This in turn motivates the identification of additional sources of heterogeneity which may be incorporated into multivariable prediction and tailoring algorithms. Moreover, quantifying the degree of variation associated with known predictors highlights variables to be controlled in clinical practice and in research. While RCTs should, in principle, produce balance over nuisance factors between treatment arms, in reality the impracticability of blinding these trials undermines this in the form of performance biases (Higgins *et al.*, 2011).

Multilevel modelling is a statistical technique that allows us to attribute variation to known and unknown factors, whilst estimating and allowing for measured covariate effects. The variation of unknown source can be apportioned to ‘between-patient’ (factors that are intrinsic to the patient) and ‘within-patient’ (factors which might vary between repeated treatment cycles) components (Snijders and Bosker, 2012). In order to investigate the impact of known and unknown sources of variation on COS response, we constructed multilevel models using a large routine ART database. We discuss the implications for the practice and research of individualized COS.

## Materials and Methods

### Population

Women aged 21–43 years undergoing ovarian stimulation for IVF (possibly with ICSI) using their own eggs at the Reproductive Medicine Department of St Mary’s Hospital, Manchester, UK, from 1 October 2008 to 8 August 2012 were included. Patients that had AMH measured using only the Gen II assay were excluded, given previously reported problems with this assay (Rustamov *et al.*, 2012). Patients with ultrasound features of polycystic ovaries, previous history of salpingectomy, ovarian cystectomy and/or unilateral salpingoophorectomy were excluded from the analysis as we expected the relationships between patient and treatment characteristics and response to be distinct in these subgroups. Similarly, small numbers of cycles with ovarian stimulation other than GnRH agonist long down regulation or short GnRH antagonist cycles were not included in the study.

Severe male factor infertility was defined as the partner having azoospermia, surgical sperm extraction or severe oligospermia, which necessitated using the multiple ejaculation resuspension and centrifugation test for assisted conception. Mild male factor was defined as abnormal sperm count that did not meet the aforementioned criteria for severe male infertility. Diagnosis of endometriosis was based on a previous history of endometriosis confirmed using laparoscopy. Diagnosis of endometrioma was established using a transvaginal ultrasound scan prior to IVF treatment. In couples without a definite cause for infertility following investigation, the diagnosis was categorized as unexplained. No patients were pretreated with oral contraceptive pill, oestrogen or progestins.

### Measurement of AMH and AFC

AMH measurements were performed by the Clinical Assay Laboratory of Central Manchester NHS Foundation Trust, and the procedure for sample handling and analysis was based on the manufacturer’s recommendations. Venous blood samples were taken without regard to the day of women’s menstrual cycle and serum samples were separated within 2 h of venipuncture. Samples were frozen at −20°C until analysed in batches using the enzymatically amplified two-site immunoassay (DSL, Active MIS/AMH ELISA; Diagnostic Systems Laboratories, Webster, TX, USA). The intra-assay coefficient of variation (CV) (*n* = 16) was 3.9% (at 10 pmol/l) and 2.9% (at 56 pmol/l). The inter-assay CV (*n* = 60) was 4.7% (at 10 pmol/l) and 4.9% (at 56 pmol/l). Haemolysed samples were not included in the study. In patients with multiple AMH measurements, the value closest to their IVF treatment cycle was selected. The working range of the assay was up to 100 pmol/l and the minimum detection limit was 0.63 pmol/l. No patients in the present study had results lower than 0.63 pmol/l. Test results that were higher than the assay range (two patients) were coded as 150% of the maximum range (150 pmol/l).

In our department, the measurement of AFC is conducted as part of an initial clinical investigation before the first consultation with clinicians and prior to the IVF cycle. Qualified radiographers performed the assessment of AFC during the early follicular phase (Day 0–5) of the menstrual cycle. Measurement of AFC consisted of the counting of all antral follicles measuring 2–6 mm in longitudinal and transverse cross sections of both ovaries using a transvaginal ultrasound scan. The AFC measurement closest to the date of the IVF cycle was selected for the analysis.

### Description of COS protocols

On the basis of their AMH measurement, patients were stratified into the treatment bands for ovarian stimulation using COS protocols. During the study, two different COS protocols were used and in addition three minor modifications were made in the second protocol. Time periods, AMH bands, down regulation regimes, initial dose of gonadotrophins and adjustment of daily dose of gonadotrophins for each protocol are described in Supplementary Table SI. Similarly, the management of excessive ovarian response was tailored to pretreatment AMH measurements, although mainly based on the results of oestradiol and scan monitoring during the stimulation period (Supplementary Table SI). Assessment of transvaginal ultrasound guided follicle tracking and serum oestradiol levels on specific days of the stimulation were used for monitoring of COS (Supplementary Table SII). The criteria for the cycle cancellation for poor ovarian response were consistent across all protocols; fewer than 3 follicles >15 mm in size on Day 10 of ovarian stimulation.

### Pituitary desensitization regimes

Selection of pituitary desensitization regime was based on the patient’s AMH according to the COH protocol at the time of commencement of the IVF cycle (Supplementary Table SI). Long agonist regimes involved daily s.c. injection of 250 μg or 500 μg of the GnRH agonist Buserelin acetate (Supercur, Sanofi Aventis Ltd., Surrey, UK) from the mid-luteal phase (Day 21) of the preceding menstrual cycle, which continued throughout ovarian stimulation. Women treated with antagonist regime had daily s.c. administration of GnRH antagonist Ganirelex (Orgalutran, Organon Laboratories Ltd., Cambridge, UK) from Day 4 post-stimulation until the day of HCG trigger. Ovarian stimulation was achieved by injection of a daily dose of hMG, Menopur (Ferring Pharmaceuticals, UK) or recombinant FSH (rFSH), Gonal F (Merck Serono, Germany) as per the AMH-tailored protocols (Supplementary Table SI). Oocyte maturation was triggered using 5000 IU HCG (Pregnyl, Organon Laboratories Ltd., Cambridge, UK) and the criteria for timing of HCG injection was consistent across all protocols: one (or more) leading follicles measuring >18 mm and two (or more) follicles >17 mm.

### Oocyte collection

Oocyte collection was conducted 34–36 h following injection of HCG for follicle maturation. An ultrasound guided oocyte pick up (OPU) was conducted by experienced clinicians under sedation. Practitioners with a small number (<10) of oocyte collection procedures were pooled in the analysis (group J). If the cycle was cancelled before oocyte recovery, it was categorized under the practitioner who was on-call for oocyte recovery on the day of cycle cancellation.

Oocytes were counted immediately post-OPU by an embryologist. In patients undergoing ICSI, the assessment of the quality of oocytes was conducted 4–6 h post-OPU. Oocytes assessed as in metaphase II stage (MII) of maturation were categorized as mature.

### Study outcomes

We evaluated that the outcomes number of oocytes recovered (IVF and ICSI cycles) and number of MII oocytes (ICSI cycles only). However, our estimates relating to MII oocytes were so imprecise as to be quite uninformative. Consequently, we present these without further comment.

### Statistical analysis

We used multilevel multivariable Poisson regression to estimate the effects of patient and treatment characteristics on stimulation response (Snijders and Bosker, 2012). The variables included in the regression models were selected on the basis of background knowledge and the objectives of the study. We distinguished patient characteristics (age, AMH, AFC, BMI, attempt number and cause of infertility) which cannot be altered from treatment variables (initial dose of gonadotrophin, stimulation regime (antagonist or long agonist), protocol (old version (v) or v1, v2 and v3 or v4 of the new protocol), type of gonadotrophin (HMG or rFSH) and OPU practitioner, which could in principle be used to tailor treatment. The representation of age, AMH and AFC in the model was determined on the basis of exploratory analysis consisting of graphing each variable against egg count and log(egg count), and by comparing models featuring competing representations using Akaike’s Information Criterion (Akaike, 1972), a measure of fit that penalizes complexity. As a result of this process, age was represented as a quadratic in the final analysis, AMH was log-transformed and AFC was categorized into three levels on the basis of quantiles. Initial dose of gonadotrophin was represented as a categorical variable; this decision was made on the basis of the distribution of the doses and the desire to obtain an easily interpretable model (Table I). Interactions between regime and other variables, and dose and other variables were considered using likelihood ratio testing and graphing of the predictors against egg count within regime and dose categories. Dose effect was allowed to vary with regime in the final analysis, owing to the observed significance of this interaction using a likelihood ratio test and the inherent plausibility of this relationship. We also fitted a version of the final model with an interaction between log(AMH) and dose, to investigate whether the relationship between dose and oocyte yield varied with AMH level. Continuous variables were mean-centred and standardized by dividing by a SD. This was done for the purposes of interpretability and to improve computational efficiency in model fitting.

**Table I:** Summary of cycle characteristics in the dataset.

Characteristic	Summary
Total dose of gonadotrophins (IU)	3000
	2100–3300
	300–7650
	*0%*
Initial dose of gonadotrophins (IU)	*0%*
75–150 IU	297 (16)
187–250 IU	484 (26)
300 IU	919 (50)
375 IU	62 (3)
450 IU	89 (5)
Age at start of cycle (years)	33.7
	30.3–36.9
	21.5–43.7
	*0%*
BMI at start of cycle (kg/m^2^)	24.0
	21.5–26.8
	16.3–36.0
	*15%*
AMH at start of cycle (pmol/l)	15.0
	9.4–22.7
	1.3–150
	*0%*
Regime	*0%*
Long Agonist	821 (44)
Antagonist	1030 (56)
Gonadotrophin	*0%*
HMG	1602
rFSH	233
AFC	13
	10–17
	3–52
	*10%*
Attempt no	*0%*
1	1347 (73)
2	409 (22)
3	91 (5)
4	4 (0)
Number of eggs recovered (cancelled cycles set to missing)	9
	5–14
	0–38
	*2%*

The dataset contained 1851 treatment cycles (defined as initiation of COS) on 1430 patients. Median, Inter-quartile range and range for continuous variables, frequency and percentage for categorical variables. %missing shown in italics. AMH, anti-Mullerian hormone; AFC, antral follicle count; rFSH, recombinant FSH.

Poisson regression models for oocyte yield and number of mature (MII) oocytes (for ICSI cycles only) as outcome variables were fitted for the final analysis with multiplicative random effects at both the observation and patient-levels included to account for the high variability in cycle outcomes and the correlation between repeated cycles undertaken by the same patient, respectively. This method produces covariate-adjusted yield ratios and 95% CIs. For categorical variables, these can be interpreted as relative yields per cycle for each level of the predictor compared to a reference category. For continuous variables, they can be interpreted as the multiplicative change in the yield per cycle associated with a SD increase in the predictor. For example, a yield ratio of two would correspond to an expected doubling of the number of oocytes obtained. We used multiple imputation to handle the relatively low proportion of missing values in the dataset (see Table I), including imputed egg counts for cancelled cycles. All of the variables included in the analysis were used in the imputation process, in addition to variables relating to follicle counts on Days 8 and 10 of the stimulation phase and the total dose of gonadotrophins administered. We examined plots of residuals and of predictions arising from the analysis to assess model fit. Analysis was conducted using the software packages R (R Core Team, 2014, Austria) and RStan (Stan Development Team, 2014, USA). Imputation was conducted using the mi package (Su *et al.*, 2011). No sample size calculation was performed, as we were not interested in hypothesis testing. Instead, we rely on 95% CIs to indicate the precision of our results. We estimated the amount of unexplained between and within-patient variation, and of total variation, in three models of oocyte yield: no covariates; patient covariates only; and treatment and patient covariates. The first of these models quantifies the variance in the data. By comparing Models 1—2, we can estimate the amount of variation attributable to patient characteristics and by comparing Models 2–3 we estimate the amount that could, in principle, be reduced through treatment. We used the distribution of the random effects from the fitted models to calculate these measures of unexplained variation. Each model yields two random effects for each patient in the analysis, which describe how each patient’s responses differ relative to the outcome that would be expected according to the model variables (patient and cycle-specific yield ratios). We calculated the yield ratio for a random effect one SD above the mean (YR_SD_), the ratio of the 95th to the 5th random effects (YR_90_), and the variance of the random effects for each model, overall and partitioned as within and between patients. YR_90_ represents the relative difference between high and low responses, after controlling for the model covariates. For example, a between-patient YR_90_ of two would indicate that, if we had two patients with the same values of the model covariates, we could reasonably expect the response of one to be double that of the other.

## Results

### Characteristics of the sample

The dataset contained 1851 treatment cycles (defined as initiation of COS) on 1430 patients. A total of 1070 (75%) patients had one cycle, 306 (21%) had two, 56 (4%) had three and one (0%) had four. Six cycles were cancelled for hyperstimulation, 86 were freeze-all cycles, and 20 were cancelled for poor response. For ICSI, 1236 cycles on 964 patients were available for the analysis of mature oocytes. Table I gives a summary of the characteristics of the cycles in the dataset.

### How much variation in COS response is explained by immutable patient characteristics?

Table II shows measures of unexplained variation (YR_SD_, YR_90_, and the residual variance, see ‘Statistical Analysis’ section) in three models of COS response.

**Table II:** Measures of unexplained variation (95% CIs) in three models of oocyte yield.

Model	Random effect YR for + 1 SD versus mean (YR_SD_)			Random effect variance			Ratio of 95th to 5th quantile of random effect YRs (YR_90_)		
	Between-patient	Within-patient	Total	Between-patient	Within-patient	Total	Between-patient	Within-patient	Total
1: No covariates	1.55	1.43	1.75	0.18	0.12	0.30	4.15	3.29	6.30
	(1.45–1.63)	(1.36–1.52)	(1.72–1.80)	(0.13–0.22)	(0.09–0.16)	(0.27–0.33)	(3.35–4.90)	(2.76–3.98)	(5.84–6.83)
2: Patient covariates (plus attempt number)	1.19	1.45	1.51	0.03	0.13	0.16	1.78	3.39	3.87
	(1.08–1.28)	(1.39–1.51)	(1.48–1.54)	(0.01–0.06)	(0.10–0.16)	(0.14–0.18)	(1.32–2.25)	(2.89–3.89)	(3.61–4.15)
3: Patient plus treatment covariates	1.23	1.36	1.45	0.04	0.10	0.13	1.98	2.70	3.36
	(1.14–1.31)	(1.30–1.42)	(1.42–1.48)	(0.02–0.07)	(0.07–0.12)	(0.12–0.15)	(1.53–2.40)	(2.31–3.16)	(3.12–3.61)

YR, Yield ratio (ratio of number of oocytes compared to the average yield).

The reduction in these measures between Models 1 and 2 tells us how much is explained by patient characteristics. It is evident that patient characteristics explain a substantial portion of the overall variation; the total unexplained variance (the sum of the between and within-patient components) reduces from 0.30 to 0.16 (i.e. to 53% of the original value) when these are added. This translates to a YR_SD_ of 1.75 in Model 1 compared to 1.51 in Model 2. The YR_90_ is 6.30 in Model 1 and 3.87 in Model 2. We can see that known patient characteristics explain variation through the between-patient rather than the within-patient component (as there is no substantive reduction in the latter, Table II). This is unsurprising, since these variables tend not to vary from cycle to cycle.

### How much variation in COS response can be explained by manipulable treatment factors?

Similarly, a comparison between Models 2 and 3 shows how much variation can be accounted for by treatment (Table II). Adding treatment variables to the model does reduce overall variation further, but only modestly. Total variance reduces from 0.16 to 0.13 (81% of the original). The YR_SD_ are 1.51 and 1.45 in the Models 2 and 3, respectively, and the YR_90_ are 3.87 and 3.36. As such, the model implies that there is a limit to the extent to which variation in response can be reduced by tailoring treatment, with the YR_90_ of 3.4 implying that a greater than three-fold difference in yield could reasonably be observed between two cycles in which two patients with similar characteristics are treated in the same way. If the same patient were to be treated in the same way on two occasions, a 2.7-fold difference in yield could reasonably be observed (YR_90_ = 2.7). This can be translated to a clinically meaningful scale. Suppose that a patient obtained nine eggs from a cycle. If another patient with similar characteristics were to be treated in the same way, we would expect their response to be between six and 13 eggs (based on YR_SD_), although any response in the range four to 19 (based on YR_2SD_) would not be surprising. If the same initial patient were stimulated in the same way a second time we would expect a response between seven and 12 eggs, but any response between five and 17 eggs should be anticipated.

### Effects of known patient and treatment characteristics

**Figure 1:**
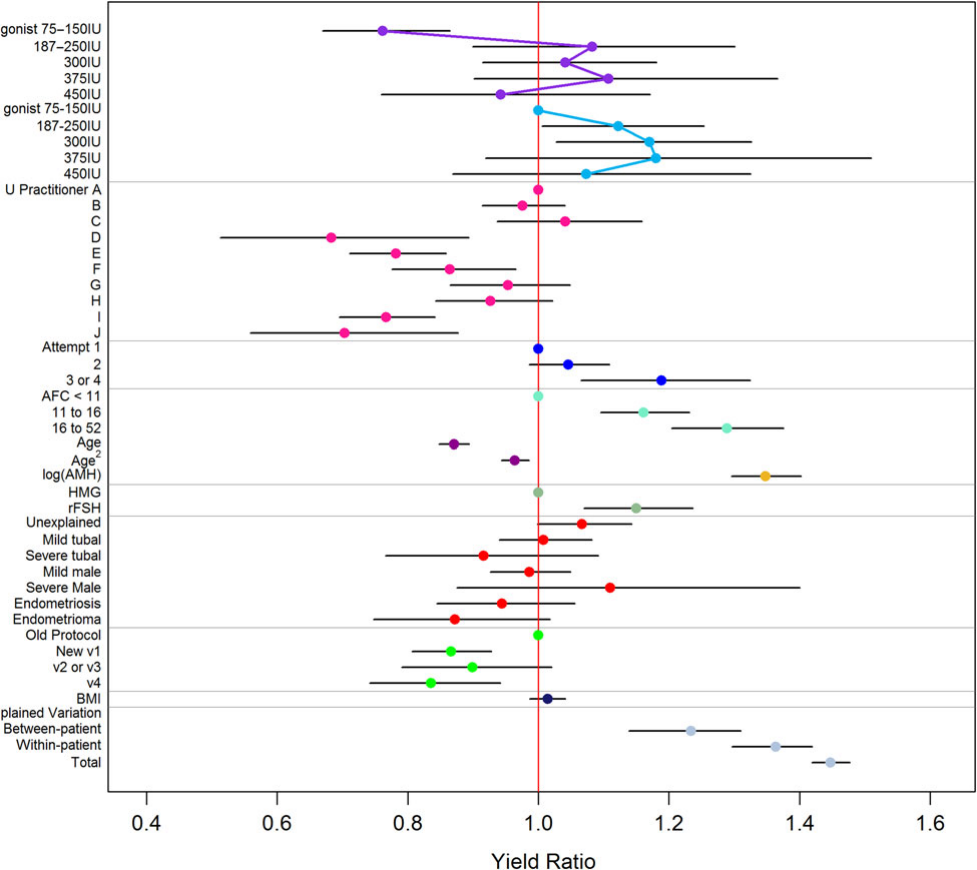
Yield ratios and 95% CIs from the multivariable Poisson regression model of number of oocytes per cycle. Continuous predictors have been standardized, so that coefficients display the expected multiplicative increase in the yield ratio for a SD change in the variable. Increasing dose effect under a GnRH antagonist regime is shown by the purple connecting line. Increasing dose effect under a GnRH long agonist regime is shown by the blue connecting line.

Yield ratios with 95% CIs from the fitted models are presented visually in Figure 1 and in Supplementary Table SIII. The corresponding estimates for the analysis of MII oocytes are displayed in Supplementary Fig. SI and Supplementary Table SIII. These refer to the estimated ‘effects’ of the predictor variables on COS response, as described in Statistical Analysis section, above. Notably, the ratio of the greatest to the lowest yield ratio estimated for the practitioners was 1.53, with differences between operators apparent on the basis of non-overlapping 95% CIs (Fig. 1). While AMH was a strong predictor of response, we did not find evidence of differential effects of AMH across dose groups (Interaction test: *P* = 0.60), although our power to detect such an effect is likely to have been low. Other predictor variables showed effects in the anticipated directions, with increased yields for higher AFC values and decreased yields for increasing age, for example. The model suggested increased yields when rFSH was used compared to an equivalent starting dose of HMG.

## Discussion

In the present study, we used multilevel modelling of a routine ART database to quantify the various sources of variation in response to COS. Our results quantify, and are consistent with, the effects of known predictors (Fig. 1, Supplementary Table SIII), while large random effects (yield ratios) suggest that there remains substantial variation that we cannot currently account for (a 3.4-fold difference Fig. 1, Table II). This holds both for differences between the responses of different women and between repeated responses of the same woman. Only a relatively small amount of this variation (around 19%) can be explained by modifiable treatment factors.

### Patient characteristics

Patient characteristics explained a substantial portion of variation between women. This included strong relationships with known measures of ovarian competence (age, AMH, AFC) (La Marca and Sunkara, 2014). The predictive value of AFC would probably be improved had it been measured at the start of each cycle, although our measurement of AFC is in line with other UK centres. A *post hoc* sensitivity analysis conducted in first attempts only (removing cycle to cycle variation) suggested a possible increase in the effect of AFC compared to our main analysis, although this was consistent with chance. Variation in BMI was quite precisely estimated as having little to no influence on oocyte yield, possibly because all patients had values in the range 19–30 kg/m^2^. It is possible that an adjustment for weight, rather than BMI, might be more meaningful, however, weight is not recorded in our database. There was no evidence to suggest that any particular infertility diagnosis was associated with number of oocytes, with the exception of increased yields (estimate of 7%, no higher than 14%) for those with unexplained infertility. Number of oocytes appeared to increase with attempt number, with increased yields for second attempts and subsequent attempts. This could reasonably be an artefact caused by selection effects relating to different profiles or treatment strategies for patients undergoing multiple treatment attempts, although a sensitivity analysis excluding attempt number had no discernible impact on the other model estimates or on the amount of explained variance.

### Treatment characteristics

This appears to be the first study to identify a substantial effect of oocyte recovery practitioner on oocyte yield. It is worth noting that the operators were all trained, experienced surgeons. While tailoring of the allocation of patients to practitioner lacks credibility as a treatment protocol, this variability does suggest that there are as yet unmeasured factors which affect COS outcome, which if identified may have the potential for optimization. This finding is important, as variation linked to recovery practitioner could undermine any attempts to guide a patient to an optimal oocyte yield by tailoring the gonadotrophin dose. Blinding of the recovery practitioner and recording of the allocation of patients to practitioner should be a mandatory feature of RCTs of personalized COS.

**Figure 2:**
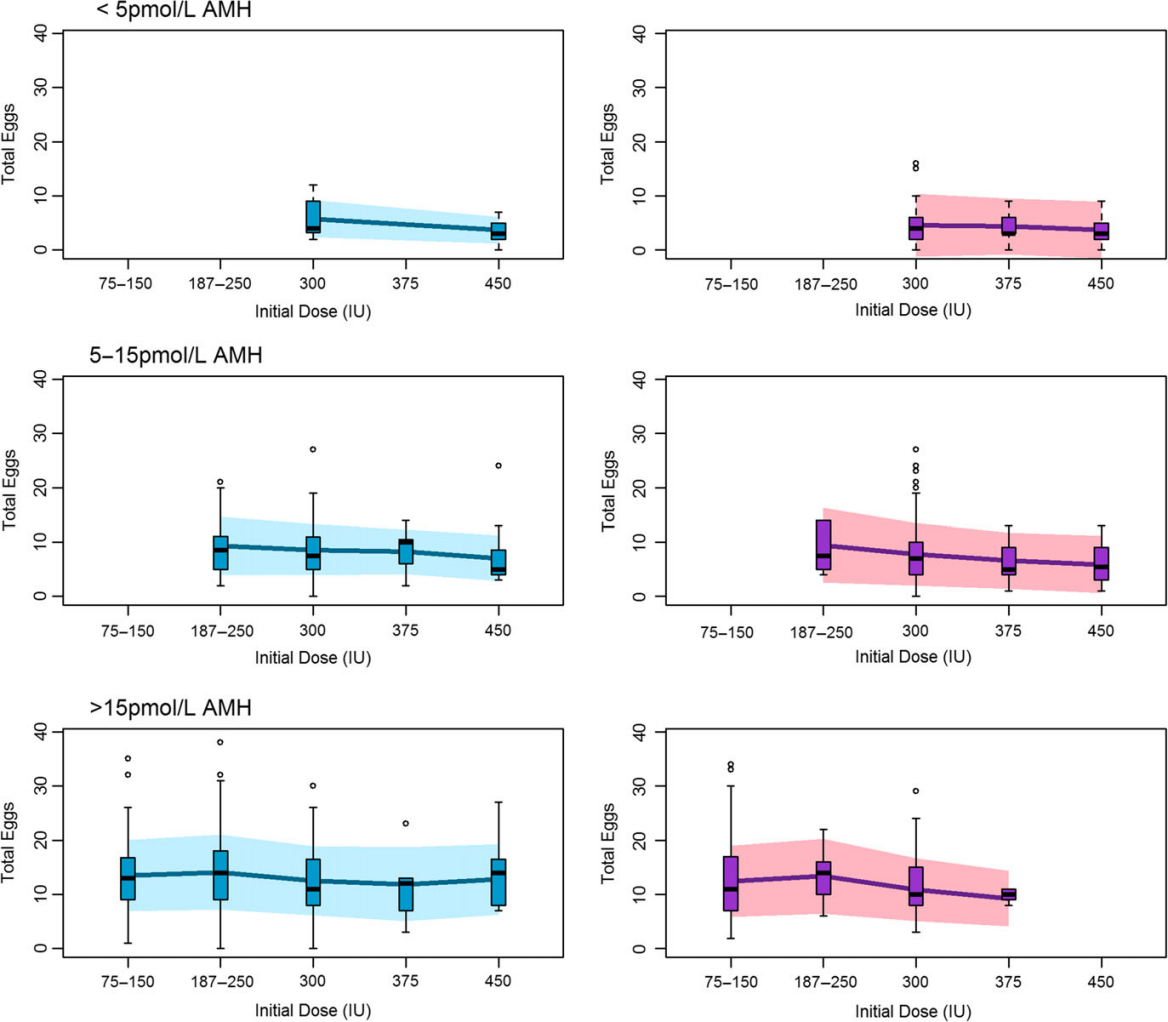
Distribution of observed egg counts. Distribution of observed egg counts (box and whisker plots) with those predicted under the model for low (DSL assay <5 pmol/l), medium (5–15 pmol/l) and high (>15 pmol/l) anti-Mullerian hormone (AMH) bands for both GnRH long agonist (blue) and GnRH antagonist (purple) regimes. Solid line represents the mean response from the posterior predictive distribution. Shaded area represents ±1 SD. Note that other covariate values are not fixed but reflect the characteristics of the sample. Only groups with five or more observations are shown.

In line with previous research in this area (Arce *et al.,* 2014), the model suggested a dose-response relationship between initial gonadotrophin dose and number of oocytes at lower doses. However, this did not appear to be sustained beyond the lowest dose. This suggests that, to the extent that tailoring the dose is possible, it should be restricted to a lower dose range (Fig. 1). Differences between antagonist and long agonist regimens were generally unclear, other than for the 75–150 IU dose band where we observed a reduced number of oocytes in antagonist cycles. In order to translate dose and regimen effects to a more easily interpretable scale, we plotted the observed oocyte yields together with the predicted oocyte yields from our model for patients falling in low, medium and high AMH bands, using cut-offs of <5, 5–15 and >15 pmol/l, which have been suggested (Nelson *et al.*, 2007) and used (Nelson *et al.*, 2009) elsewhere in the literature (Fig. 2). This represents the predicted outcomes for our centre, were dose selection performed solely on the basis of AMH. Figure 2 highlights the impact of other sources of variation that should be considered in individualized COS, because the variation within each AMH/protocol/dose category is large relative to the variation between categories, and suggests that multivariable algorithms (Popovic-Todorovic *et al.*, 2003, La Marca *et al.*, 2012) will be needed to obtain reliable predictions of response. However, our models also suggest that many of these contributory variables remain unknown. We did not replicate the finding of Arce *et al.* (2014) that dose effects vary according to AMH, although our power to detect an effect of this nature is likely to have been low. The predictions appear to be consistent with existing research and writing on this topic, indicating in particular that increasing the dose in patients with predicted low response is unlikely to increase the oocyte yield (Klinkert *et al.*, 2005, Lekamge *et al.*, 2008) and that dose-effects on the mean response are modest (Sterrenburg *et al.*, 2011).

In this case, the effect on the mean response may not represent the most useful measure of efficacy however. Given that the goal of individualized COS is to prevent insufficient or unsafe responses (La Marca *et al.*, 2012), we believe that it is most useful to focus on the effects of interventions on reducing variation in outcome. In this context, an intervention could be ‘effective’ even if no effect on the mean was observed. Our analysis suggests that treatment differences account for relatively little of this variation, and this is likely to limit the extent to which extreme responses can be prevented by tailoring treatment. A unidirectional mean effect will of course be more relevant in populations of expected poor or high responders compared to unselected patients, although even then a simple ‘mean difference’ may conceal deleterious consequences of treatment (if, for example, more expected high (low) responders end up having poor (excessive) responses, as appears to be the case in Nyboe Andersen *et al.*, 2017). As a result, many trials quantify COS response by categorizing responses as ‘poor’,’normal’ or ‘high’, and use this as a trial endpoint (eg: Allegra *et al.*, 2017; Popovic-Todorovic *et al.*, 2003). This is not entirely unreasonable if the criteria are predefined and cancelled cycles are included in the denominator, although categorizing measurements in this way reduces power in the trial, necessitating larger sample sizes (Altman and Royston, 2006). We note that simple statistical methods exist for comparing variation between treatment arms directly, such as Levene’s test (Schultz, 1985).

Limitations of the present study should be noted. There may be concerns over the generalizability of our findings, since some of the doses administered in the dataset are higher than would typically be used, for example, throughout Europe. However, we note here that our concern is not in the evaluation of any particular treatment strategy, but rather to tease apart the contributions of various predictors on COS response. Regardless, we conducted a sensitivity analysis where we fitted a model in the subset of participants treated with doses of 225 IU or lower (Supplementary Fig. S2). The estimates are consistent with our main analysis, albeit with reduced precision due to the reduction in sample size. While we included a large number of predictor variables, there is likely to be confounding due to unmeasured predictors as well as ‘residual confounding’ due to measurement error in the model covariates (Sterne *et al.*, 2016). In particular, there may be concern around confounding by indication due to selection for treatment on the basis of prognosis (Walker, 1996). In this regard, we note that we have included all of the variables that were used for treatment allocation in the model (at least in principle), and measures of balance between dose groups (McCaffrey *et al.*, 2013) suggest a reasonable degree of balance after adjusting for covariates, other than for the highest versus the lowest dose band. In addition, there are plausible sources of variation which we have not been able to incorporate into the model. For example, rFSH was initiated between Days 2 and 4 in GnRH antagonist cycles, and the exact start day might explain some of the variation. Another example would be the length of the menstrual cycle. We note that the predictive value would have to be large to substantively change our conclusions regarding the scope for individualized treatment. Nonetheless, it remains to quantify these possible sources of variation. Total dose and stimulation duration were not included in the model. This is because these factors are largely determined by the patient response, as observed on ultrasound. Total dose is effectively a surrogate outcome for anticipated stimulation response; dose is increased when things are going poorly. When we include total dose as a covariate, for example, the estimate is negative (Supplementary Table SIV). It would, therefore, constitute a statistical error to include these variables in the model, as to do so would be to adjust for some of the response. We have instead included the pre-treatment variable ‘protocol’, which captures policy changes in relation to stimulation. This will, of course, be subject to measurement error arising from variation in protocol adherence. Although, we present a sensitivity analysis including total dose and stimulation duration in Supplementary Table SIV, we would stress that we do not believe that this model is interpretable.

We suggest that an understanding of the degree and determinants of variation in COS response is key to improving clinical practice and conducting research in this area.

The goal of personalized COS is to reduce this variation, and this may be assisted both by incorporating a range of predictive patient characteristics into dose algorithms and by attempting to standardize aspects of treatment that may introduce noise (Senn, 2016). Our results indicate that much of the variation in response cannot be explained by known factors however. We have identified the oocyte recovery practitioner as one potential source of variation in this study, and recommend that blinding is used in RCTs to reduce associated performance biases. Moreover, we advise that the allocation of participant to practitioner is recorded and considered as a covariate in any analysis. We conclude that, until additional predictors of variation are identified, consistent prevention of extreme responses is unlikely to be achieved.

**Supplementary Table SI:** AMH-tailored stratification protocols for regime, starting dose of hMG/rFSH and adjusting daily dose of gonadotrophins (St Mary’s Hospital).

Protocol 1	Protocol 2 (V1)	Protocol 2 (v2)	Protocol 2 (v3)	Protocol 2 (v4)
(01 September 2008–31 December 2010)	(01 January 2011–30 April 2011)	(01 May 2011–31 July 2011)	(01 August 2011–30 November 2011)	(01 December 2011-08 August 2012)
**Initial dose (Day 1**–**3)**	**Initial dose (Day 1**–**3)**	**Initial dose (Day 1**–**3)**	**Initial dose (Day 1**–**3)**	**Initial dose (Day 1**–**3)**
<**2.2 AMH (DSL)** Exclude **2.2**–**15.6 AMH (DSL)** Antagonist: 300 hMG **15.7**–**28.5 AMH (DSL)** *Long Agonist*: *200 rFSH/225 hMG* >**28.6 AMH (DSL)** Antagonist: 150 hMG	<**3 AMH (Gen II)** Co-Flare: 450 hMG **3**–**10 AMH (Gen II)** Antagonist: 375 hMG **11**–**21 AMH (Gen II)** Long Agonist: 300 hMG **22**–**30 AMH (Gen II)** Long Agonist: 225 hMG **31**–**39 AMH (Gen II)** Long Agonist: 150 hMG **40**–**67 AMH (Gen II)** without PCO Long Agonist: 150 hMG **40**–**67 AMH (Gen II)** with PCO *Long Agonist: 125 rFSH* >**67 AMH (Gen II)** Long *Agonist: 112.5 rFSH*	<**3 AMH (Gen II)** Co-Flare: 450 hMG **3**–**10 AMH (Gen II)** Antagonist: 300 hMG **11**–**21 AMH (Gen II)** Long Agonist: 225 hMG **22**–**39 AMH (Gen II)** **without PCOS** Long Agonist: 150 hMG **22**–**39 AMH (Gen II)** **with PCOS** Long Agonist: 150 rFSH **40**–**67 AMH (Gen II)** **without PCOS** Long Agonist: 150 hMG **40**–**67 AMH (Gen II)** **with PCOS** Long Agonist: 112.5 rFSH >**67 AMH (Gen II)** Long Agonist: 112.5 rFSH	**2**–**3 AMH (Gen II)** Antagonist: 450 hMG **3**–**10 AMH (Gen II)** Long Agonist: 300 hMG **11**–**21 AMH (Gen II)** Long Agonist: 225 hMG **22**–**39 AMH (Gen II)** **without PCOS** Long Agonist: 150 hMG **22**–**39 AMH (Gen II)** **with PCOS** Antagonist: 150 rFSH **40**–**67 AMH (Gen II)** without PCOS Antagonist: 150 hMG **40**–**67 AMH (Gen II)** **with PCOS** Antagonist: 112.5 rFSH >**67 AMH (Gen II)** Antagonist: 112.5 rFSH	**2**–**3 AMH (Gen II)** Antagonist: 300 rFSH **3**–**10 AMH (Gen II)** Long Agonist: 225 rFSH **11**–**21 AMH (Gen II)** Long Agonist: 187.5 rFSH **22**–**39 AMH (Gen II)** **without PCOS** Long Agonist: 150 hMG **22**–**39 AMH (Gen II)** **with PCOS** Antagonist: 150 hMG **40**–**67 AMH (Gen II)** **without PCOS** Antagonist: 150 hMG **40**–**67 AMH (Gen II)** **with PCOS** Antagonist: 112.5 hMG >**67 AMH (Gen II)** Antagonist: 112.5 hMG
**Dose adjustment**	**Dose adjustment**	**Dose adjustment**	**Dose adjustment**	**Dose adjustment**
*No or minimum change on daily dose of gonadotrophin*	*Step up or down using Oestradiol levels (Day 3&6) and Ultrasound follicle tracking (Day 8)*	*Step up or down using Oestradiol levels (Day 3&6) and Ultrasound follicle tracking (Day 8)*	*Step up or down using Oestradiol levels (Day 3&6) and Ultrasound follicle tracking (Day 8)*	*Step up or down using Oestradiol levels (Day 3&6) and Ultrasound follicle tracking (Day 8)*

**Supplementary Table SII:** AMH-tailored stratification protocols for management of suspected excessive response (St Mary’s Hospital).

	Protocol 1 (01 September 2008–2031 December 2010)	Protocol 2 (v1) (01 January 2011–2030 April 2011) and Protocol 2 (v2) (01 May 2011–31 July 2011)	Protocol 2 (v3) (01 August 2011–30 November 2011)	Protocol 2 (v4) (01 December 2011-2008 August 2012)
Coasting for excessive response on Day 8	Oestradiol >20 000 pg/ml	30–40 follicles larger than 10 mm or	30–40 follicles larger than 12 mm	No coasting
		Oestradiol >18 000 pg/ml		
Coasting for excessive response once follicle maturation meets criteria	Oestradiol >20 000 pg/ml	30–40 follicles larger than 10 mm	25–40 follicles larger than 10 mm	25–30 follicles larger than 15 mm
	Day 8 or thereafter	Day 8 or thereafter	Day 10 or thereafter	Day 8 or thereafter
Cancellation for excessive response	Oestradiol l >20 000 pg/ml and symptoms of OHSS after >3 days of coasting	More than 40 follicles larger than 10 mm	More than 40 follicles larger than 15 mm	Cancel only if symptoms of OHSS

**Supplementary Table SIII:** Yield ratios and 95% CIs from fitted Poisson regression models of number of oocytes and of number of mature oocytes, with the covariates shown in the table. Estimates for treatment characteristics relate to total effects after holding patient characteristics fixed. Estimates for patient characteristics relate to direct effects on COS response (ie: after subtracting the ‘effect’ of characteristics on treatment selection).

Parameter	Number of oocytes	Number of MII oocytes
Intercept	8.91 (7.79–10.22)	7.14 (5.27–9.64)
Treatment characteristics		
Long Agonist 75–150 IU	1.00	1.00
Long Agonist 187–250 IU	1.12 (1.01–1.25)	1.02 (0.83–1.24)
Long Agonist 300 IU	1.17 (1.03–1.33)	1.14 (0.90–1.43)
Long Agonist 375 IU	1.18 (0.92–1.51)	1.01 (0.67–1.55)
Long Agonist 450 IU	1.07 (0.87–1.33)	0.83 (0.58–1.20)
Antagonist 75–150 IU	0.76 (0.67–0.86)	0.76 (0.61–0.96)
Antagonist 187–250 IU	1.08 (0.90–1.30)	1.19 (0.86–1.67)
Antagonist 300 IU	1.04 (0.91–1.18)	0.98 (0.78–1.23)
Antagonist 375 IU	1.11 (0.90–1.37)	1.30 (0.90–1.88)
Antagonist 450 IU	0.94 (0.76–1.17)	0.91 (0.63–1.33)
OPU operator: A	1.00	1.00
B	0.98 (0.91–1.04)	0.90 (0.79–1.01)
C	1.04 (0.94–1.16)	1.03 (0.85–1.24)
D	0.68 (0.51–0.89)	0.79 (0.47–1.37)
E	0.78 (0.71–0.86)	0.85 (0.73–1.00)
F	0.86 (0.78–0.97)	0.77 (0.62–0.97)
G	0.95 (0.87–1.05)	0.91 (0.76–1.09)
H	0.93 (0.84–1.02)	0.94 (0.78–1.12)
I	0.77 (0.70–0.84)	0.83 (0.70–0.98)
J	0.70 (0.56–0.88)	0.56 (0.35–0.91)
Protocol: Old	1.00	1.00
New protocol (V1)	0.87 (0.81–0.93)	0.89 (0.79–1.01)
New protocol (V2 and V3)	0.90 (0.79–1.02)	0.99 (0.79–1.24)
New protocol (V4)	0.84 (0.74–0.94)	0.85 (0.68–1.06)
Patient characteristics		
Attempt No:1st	1.00	1.00
2nd	1.05 (0.99–1.11)	1.03 (0.92–1.15)
3rd or 4th	1.19 (1.07–1.32)	1.08 (0.90–1.29)
Antral follicle count: <10	1.00	1.00
11–16	1.16 (1.11–1.23)	1.14 (1.01–1.27)
16–52	1.29 (1.20–1.38)	1.22 (1.07–1.38)
Age (SDs)	0.87 (0.85–0.89)	0.91 (0.87–0.96)
Age^2^ (SDs)	0.96 (0.94–0.99)	0.96 (0.93–1.00)
Log (AMH) (SDs)	1.35 (1.30–1.40)	1.29 (1.21–1.38)
Gonadotropin: HMG	1.00	1.00
rFSH	1.15 (1.07–1.24)	1.13 (0.99–1.29)
Unexplained fertility	1.07 (1.00–1.14)	1.03 (0.91–1.17)
Mild tubal	1.01 (0.94–1.08)	0.96 (0.85–1.10)
Severe tubal	0.92 (0.77–1.09)	0.92 (0.66–1.30)
Mild male factor	0.99 (0.93–1.05)	1.02 (0.92–1.13)
Severe male factor	1.11 (0.88–1.40)	0.96 (0.64–1.44)
Endometriosis	0.94 (0.85–1.06)	0.89 (0.72–1.12)
Endometrioma	0.87 (0.75–1.02)	0.89 (0.68–1.18)
BMI (SDs)	1.01 (0.99–1.04)	1.00 (0.96–1.05)

**Supplementary Table SIV:** Yield ratios and 95% CIs from fitted Poisson regression model of number of oocytes with the covariates shown in the table. The estimates do not have a clear interpretation, because total dose and stimulation duration are partially determined by response.

Parameter	Yield ratio	95% CI	
Intercept	1.81	1.31	2.5
Age (SDs)	0.88	0.85	0.91
Age^2^	0.97	0.94	0.99
LDR: 75-150IU	1		
LDR: 187-250IU	1.31	1.15	1.49
LDR: 300IU	1.48	1.27	1.73
LDR: 375IU	1.72	1.29	2.31
LDR: 450IU	1.59	1.21	2.09
Ant:75-150IU	0.80	0.69	0.91
Ant: 187-250IU	1.28	1.05	1.58
Ant: 300IU	1.22	1.05	1.41
Ant: 375IU	1.51	1.19	1.93
Ant:450IU	1.54	1.18	2.02
BMI (SDs)	1.01	1	1.01
log(AMH) (SDs)	1.46	1.37	1.56
Gonadotropin: HMG	1		
rFSH	1.08	1	1.18
AFC: <10			
11–16	1.16	1.09	1.24
16–52	1.29	1.21	1.39
OPU A	1		
OPU B	1.01	0.74	1.38
OPU C	1.29	1.12	1.48
OPU D	1.28	1.13	1.46
OPU E	0.82	0.63	1.08
OPU F	1.31	1.17	1.45
OPU G	1.4	1.22	1.62
OPU H	1.33	1.2	1.47
OPU I	1.16	1.00	1.35
OPU J	1.08	0.95	1.23
Unexplained	1.04	0.97	1.12
mtubal	0.99	0.92	1.07
stubal	0.96	0.8	1.15
mm	0.96	0.9	1.03
sm	1.13	0.88	1.45
endometriosis	0.94	0.84	1.07
endometrioma	0.91	0.78	1.06
Total dose of gonadotropins (SDs)	0.84	0.79	0.89
Stimulation duration (SDs)	1.13	1.09	1.18
Old protocol	1		
New Protocol: V1	0.84	0.78	0.91
V2 & V3	0.85	0.74	0.98
V4	0.81	0.71	0.93

## Supplementary Material

Supplementary Data

Supplementary Data

Supplementary Data

Supplementary Data

Supplementary Data

Supplementary Data

## References

[hox018C1] Akaike H Information theory and an extension of the maximum likelihood principle. In: *Proc. 2nd Int. Symp. Information Theory, Supp. to Problems of Control and Information Theory*, 1972, 267–281.

[hox018C2] Allegra A, Marino A, Volpes A, Coffaro F, Scaglione P, Gullo S, La Marca A A randomized controlled trial investigating the use of a predictive nomogram for the selection of the FSH starting dose in IVF/ICSI cycles. Reprod Biomed Online 2017;34:429–438. 28189417 10.1016/j.rbmo.2017.01.012

[hox018C3] Altman DG, Royston P The cost of dichotomising continuous variables. BMJ 2006;332:1080. 16675816 10.1136/bmj.332.7549.1080 PMC1458573

[hox018C4] Arce JC, Andersen AN, Fernandez-Sanchez M, Visnova H, Bosch E, Garcia-Velasco JA, Barri P, De Sutter P, Klein BM Fauser BCJM. Ovarian response to recombinant human follicle-stimulating hormone: a randomized, antimullerian hormone-stratified, dose-response trial in women undergoing in vitro fertilization/intracytoplasmic sperm injection. Fertil Steril 2014;102:1633–U1456. 25256937 10.1016/j.fertnstert.2014.08.013

[hox018C5] Broer SL, Dolleman M, Opmeer BC, Fauser BC, Mol BW, Broekmans FJM AMH and AFC as predictors of excessive response in controlled ovarian hyperstimulation: a meta-analysis. Hum Reprod Update 2011;17:46–54. 20667894 10.1093/humupd/dmq034

[hox018C6] Broer SL, Dolleman M, van Disseldorp J, Broeze KA, Opmeer BC, Bossuyt PMM, Eijkemans MJC, Mol BW, Broekmans FJM, Grp et al Prediction of an excessive response in in vitro fertilization from patient characteristics and ovarian reserve tests and comparison in subgroups: an individual patient data meta-analysis. Fertil Steril 2013;100:420–429. 23721718 10.1016/j.fertnstert.2013.04.024

[hox018C7] Higgins JP, Altman DG, Gotzsche PC, Juni P, Moher D, Oxman AD, Savovic J, Schulz KF, Weeks L, Sterne JA et al The Cochrane Collaboration’s tool for assessing risk of bias in randomised trials. BMJ 2011;343:d5928. 22008217 10.1136/bmj.d5928 PMC3196245

[hox018C8] Klinkert E, Velde ET, Broekmans F ‘Defining poor ovarian response during IVF cycles, in women aged <40 years, and its relationship with treatment outcome’. Hum Reprod 2005;20:573–573. 15670974 10.1093/humrep/deh622

[hox018C9] Kurinczuk JJH, C. Fertility Treatment in 2006 - a statistical analysis. *Human Fertilization and Embryology Authority* 2010; London.

[hox018C10] La Marca A, Papaleo E, Grisendi V, Argento C, Giulini S, Volpe A Development of a nomogram based on markers of ovarian reserve for the individualisation of the follicle-stimulating hormone starting dose in in vitro fertilisation cycles. BJOG 2012;119:1171–1179. 22805536 10.1111/j.1471-0528.2012.03412.x

[hox018C11] La Marca A, Sunkara SK Individualization of controlled ovarian stimulation in IVF using ovarian reserve markers: from theory to practice. Hum Reprod Update 2014;20:124–140. 24077980 10.1093/humupd/dmt037

[hox018C12] Lekamge DN, Lane M, Gilchrist RB, Tremellen KP Increased gonadotrophin stimulation does not improve IVF outcomes in patients with predicted poor ovarian reserve. J Assist Reprod Genet 2008;25:515–521. 18972201 10.1007/s10815-008-9266-6 PMC2593769

[hox018C13] McCaffrey DF, Griffin BA, Almirall D, Slaughter ME, Ramchand R, Burgette LF A tutorial on propensity score estimation for multiple treatments using generalized boosted models. Stat Med 2013;32:3388–3414. 23508673 10.1002/sim.5753 PMC3710547

[hox018C14] Nelson SM, Yates RW, Fleming R Serum anti-Mullerian hormone and FSH: prediction of live birth and extremes of response in stimulated cycles—implications for individualization of therapy. Hum Reprod 2007;22:2414–2421. 17636277 10.1093/humrep/dem204

[hox018C15] Nelson SM, Yates RW, Lyall H, Jamieson M, Traynor I, Gaudoin M, Mitchell P, Ambrose P, Fleming R Anti-Mullerian hormone-based approach to controlled ovarian stimulation for assisted conception. Hum Reprod 2009;24:867–875. 19136673 10.1093/humrep/den480

[hox018C16] Nyboe Andersen A, Nelson SM, Fauser BC, Garcia-Velasco JA, Klein BM, Arce JC, group E-s Individualized versus conventional ovarian stimulation for in vitro fertilization: a multicenter, randomized, controlled, assessor-blinded, phase 3 noninferiority trial. Fertil Steril 2017;107:387–396 e384. 27912901 10.1016/j.fertnstert.2016.10.033

[hox018C17] Popovic-Todorovic B, Loft A, Bredkjaeer HE, Bangsboll S, Nielsen IK, Andersen AN A prospective randomized clinical trial comparing an individual dose of recombinant FSH based on predictive factors versus a ‘standard’ dose of 150 IU/day in ‘standard’ patients undergoing IVF/ICSI treatment. Hum Reprod 2003;18:2275–2282. 14585873 10.1093/humrep/deg472

[hox018C18] R Core Team R: A Language and Environment for Statistical Computing. Vienna, Austria: R Foundation for Statistical Computing, 2014.

[hox018C19] Rustamov O, Smith A, Roberts SA, Yates AP, Fitzgerald C, Krishnan M, Nardo LG, Pemberton PW Anti-Mullerian hormone: poor assay reproducibility in a large cohort of subjects suggests sample instability. Hum Reprod 2012;27:3085–3091. 22777530 10.1093/humrep/des260

[hox018C20] Schultz BB Levene test for relative variation. Syst Zool 1985;34:449–456.

[hox018C21] Senn S Mastering variation: variance components and personalised medicine. Stat Med 2016;35:966–977. 26415869 10.1002/sim.6739 PMC5054923

[hox018C22] Snijders TAB, Bosker RJ Multilevel Analysis: An Introduction to Basic and Advanced Multilevel Modeling, 2nd edn Los Angeles; London: SAGE, 2012.

[hox018C23] Stan Development Team RStan: the R interface to Stan, Version 2.5.0. 2014.

[hox018C24] Sterne JAC, Hernan MA, Reeves BC, Savovic J, Berkman ND, Viswanathan M, Henry D, Altman DG, Ansari MT, Boutron I et al ROBINS-I: a tool for assessing risk of bias in non-randomised studies of interventions. Bmj-Brit Med J 2016;355:i4919. 10.1136/bmj.i4919 PMC5062054 27733354

[hox018C25] Sterrenburg MD, Veltman-Verhulst SM, Eijkemans MJC, Hughes EG, Macklon NS, Broekmans FJ Fauser BCJM. Clinical outcomes in relation to the daily dose of recombinant follicle-stimulating hormone for ovarian stimulation in in vitro fertilization in presumed normal responders younger than 39 years: a meta-analysis. Hum Reprod Update 2011;17:184–196. 20843965 10.1093/humupd/dmq041

[hox018C26] Steward RG, Lan L, Shah AA, Yeh JS, Price TM, Goldfarb JM, Muasher SJ Oocyte number as a predictor for ovarian hyperstimulation syndrome and live birth: an analysis of 256,381 in vitro fertilization cycles. Fertil Steril 2014;101:967–973. 24462057 10.1016/j.fertnstert.2013.12.026

[hox018C27] Su YS, Gelman A, Hill J, Yajima M Multiple imputation with diagnostics (mi) in R: opening windows into the black box. J Stat Softw 2011;45:1–31.

[hox018C28] Sunkara SK, La Marca A, Seed PT, Khalaf Y Increased risk of preterm birth and low birthweight with very high number of oocytes following IVF: an analysis of 65 868 singleton live birth outcomes. Hum Reprod 2015;30:1473–1480. 25883033 10.1093/humrep/dev076

[hox018C29] Sunkara SK, Rittenberg V, Raine-Fenning N, Bhattacharya S, Zamora J, Coomarasamy A Association between the number of eggs and live birth in IVF treatment: an analysis of 400 135 treatment cycles. Hum Reprod 2011;26:1768–1774. 21558332 10.1093/humrep/der106

[hox018C30] van Tilborg TC, Broekmans FJM, Dolleman M, Eijkemans MJC, Mol B, Laven JSE, Torrance HL Individualized follicle-stimulating hormone dosing and in vitro fertilization outcome in agonist downregulated cycles: a systematic review. Acta Obstet Gynecol Scand 2016;95:1333–1344. 27687487 10.1111/aogs.13032

[hox018C31] Walker AM Confounding by indication. Epidemiology 1996;7:335–336. 8793355

