# Supplementary material for: How much variation in oocyte yield after controlled ovarian stimulation can be explained? A multilevel modelling study
Source: Hum Reprod Open. 2017 Nov 13;2017(3):hox018. doi: 10.1093/hropen/hox018 (PMC6276674; doi:10.1093/hropen/hox018)
Supplement: Supplementary Data [file hox018suppl_table1.pdf]

Supplementary Table SI **AMH-tailored stratification protocols for regime, starting dose of hMG/rFSH and adjusting daily dose of gonadotrophins (St Mary's Hospital).**

**Protocol 1 (01 September 2008–31 December 2010)**	**Protocol 2 (V1) (01 January 2011–30 April 2011)**	**Protocol 2 (v2) (01 May 2011–31 July 2011)**	**Protocol 2 (v3) (01 August 2011–30 November 2011)**	**Protocol 2 (v4) (01 December 2011-08 August 2012)**
**Initial dose (Day 1–3)**	**Initial dose (Day 1–3)**	**Initial dose (Day 1–3)**	**Initial dose (Day 1–3)**	**Initial dose (Day 1–3)**
(1) **<2.2 AMH (DSL)**<br>*Exclude*<br>(2) **2.2–15.6 AMH (DSL)**<br>*Antagonist: 300 hMG*<br>(3) **15.7–28.5 AMH (DSL)**<br>*Long Agonist: 200 rFSH/225 hMG*<br>(4) **>28.6 AMH (DSL)**<br>*Antagonist: 150 hMG*	(1) **<3 AMH (Gen II)**<br>*Co-Flare: 450 hMG*<br>(2) **3–10 AMH (Gen II)**<br>*Antagonist: 375 hMG*<br>(3) **11–21 AMH (Gen II)**<br>*Long Agonist: 300 hMG*<br>(4) **22–30 AMH (Gen II)**<br>*Long Agonist: 225 hMG*<br>(5) **31–39 AMH (Gen II)**<br>*Long Agonist: 150 hMG*<br>(6) **40–67 AMH (Gen II)**<br>without PCO<br>Long Agonist: 150 hMG<br>(7) **40–67 AMH (Gen II)**<br>with PCO *Long Agonist: 125 rFSH*<br>(8) **>67 AMH (Gen II)**<br>Long Agonist: *112.5 rFSH*	(1) **<3 AMH (Gen II)**<br>*Co-Flare: 450 hMG*<br>(2) **3–10 AMH (Gen II)**<br>*Antagonist: 300 hMG*<br>(3) **11–21 AMH (Gen II)**<br>*Long Agonist: 225 hMG*<br>(4) **22–39 AMH (Gen II) without PCOS**<br>*Long Agonist: 150 hMG*<br>(5) **22–39 AMH (Gen II) with PCOS**<br>*Long Agonist: 150 rFSH*<br>(6) **40–67 AMH (Gen II) without PCOS**<br>*Long Agonist: 150 hMG*<br>(7) **40–67 AMH (Gen II) with PCOS**<br>*Long Agonist: 112.5 rFSH*<br>(8) **>67 AMH (Gen II)**<br>*Long Agonist: 112.5 rFSH*	(1) **2–3 AMH (Gen II)**<br>*Antagonist: 450 hMG*<br>(2) **3–10 AMH (Gen II)**<br>*Long Agonist: 300 hMG*<br>(3) **11–21 AMH (Gen II)**<br>*Long Agonist: 225 hMG*<br>(4) **22–39 AMH (Gen II) without PCOS**<br>*Long Agonist: 150 hMG*<br>(5) **22–39 AMH (Gen II) with PCOS**<br>*Antagonist: 150 rFSH*<br>(6) **40–67 AMH (Gen II)**<br>without PCOS<br>*Antagonist: 150 hMG*<br>(7) **40–67 AMH (Gen II) with PCOS**<br>*Antagonist: 112.5 rFSH*<br>(8) **>67 AMH (Gen II)**<br>*Antagonist: 112.5 rFSH*	(1) **2–3 AMH (Gen II)**<br>*Antagonist: 300 rFSH*<br>(2) **3–10 AMH (Gen II)**<br>*Long Agonist: 225 rFSH*<br>(3) **11–21 AMH (Gen II)**<br>*Long Agonist: 187.5 rFSH*<br>(4) **22–39 AMH (Gen II) without PCOS**<br>*Long Agonist: 150 hMG*<br>(5) **22–39 AMH (Gen II) with PCOS**<br>*Antagonist: 150 hMG*<br>(6) **40–67 AMH (Gen II) without PCOS**<br>*Antagonist: 150 hMG*<br>(7) **40–67 AMH (Gen II) with PCOS**<br>*Antagonist: 112.5 hMG*<br>(8) **>67 AMH (Gen II)**<br>*Antagonist: 112.5 hMG*
**Dose adjustment**	**Dose adjustment**	**Dose adjustment**	**Dose adjustment**	**Dose adjustment**
No or minimum change on daily dose of gonadotrophin	*Step up or down using Oestradiol levels (Day 3&6) and Ultrasound follicle tracking (Day 8)*	*Step up or down using Oestradiol levels (Day 3&6) and Ultrasound follicle tracking (Day 8)*	*Step up or down using Oestradiol levels (Day 3&6) and Ultrasound follicle tracking (Day 8)*	*Step up or down using Oestradiol levels (Day 3&6) and Ultrasound follicle tracking (Day 8)*
