# Supplementary material for: How much variation in oocyte yield after controlled ovarian stimulation can be explained? A multilevel modelling study
Source: Hum Reprod Open. 2017 Nov 13;2017(3):hox018. doi: 10.1093/hropen/hox018 (PMC6276674; doi:10.1093/hropen/hox018)
Supplement: Supplementary Data [file hox018suppl_table2.pdf]

Supplementary Table SII **AMH-tailored stratification protocols for management of suspected excessive response (St Mary's Hospital).**

	Protocol 1 (01 September 2008–2031 December 2010)	Protocol 2 (v1) (01 January 2011–2030 April 2011) and Protocol 2 (v2) (01 May 2011–31 July 2011)	Protocol 2 (v3) (01 August 2011–30 November 2011)	Protocol 2 (v4) (01 December 2011-2008 August 2012)
Coasting for excessive response on Day 8	Oestradiol >20 000 pg/ml	30–40 follicles larger than 10 mm or	30–40 follicles larger than 12 mm	No coasting
		Oestradiol >18 000 pg/ml		
Coasting for excessive response once follicle maturation meets criteria	Oestradiol >20 000 pg/ml	30–40 follicles larger than 10 mm	25–40 follicles larger than 10 mm	25–30 follicles larger than 15 mm
	Day 8 or thereafter	Day 8 or thereafter	Day 10 or thereafter	Day 8 or thereafter
Cancellation for excessive response	Oestradiol l >20 000 pg/ml and symptoms of OHSS after >3 days of coasting	More than 40 follicles larger than 10 mm	More than 40 follicles larger than 15 mm	Cancel only if symptoms of OHSS
