# Supplementary material for: How much variation in oocyte yield after controlled ovarian stimulation can be explained? A multilevel modelling study
Source: Hum Reprod Open. 2017 Nov 13;2017(3):hox018. doi: 10.1093/hropen/hox018 (PMC6276674; doi:10.1093/hropen/hox018)
Supplement: Supplementary Data [file hox018suppl_table3.pdf]

Supplementary Table SIII **Yield ratios and 95% CIs from fitted Poisson regression models of number of oocytes and of number of mature oocytes, with the covariates shown in the table. Estimates for treatment characteristics relate to total effects after holding patient characteristics fixed. Estimates for patient characteristics relate to direct effects on COS response (ie: after subtracting the 'effect' of characteristics on treatment selection).**

Parameter	Number of oocytes	Number of MII oocytes
Intercept	8.91 (7.79–10.22)	7.14 (5.27–9.64)
Treatment characteristics		
Long Agonist 75–150 IU	1.00	1.00
Long Agonist 187–250 IU	1.12 (1.01–1.25)	1.02 (0.83–1.24)
Long Agonist 300 IU	1.17 (1.03–1.33)	1.14 (0.90–1.43)
Long Agonist 375 IU	1.18 (0.92–1.51)	1.01 (0.67–1.55)
Long Agonist 450 IU	1.07 (0.87–1.33)	0.83 (0.58–1.20)
Antagonist 75–150 IU	0.76 (0.67–0.86)	0.76 (0.61–0.96)
Antagonist 187–250 IU	1.08 (0.90–1.30)	1.19 (0.86–1.67)
Antagonist 300 IU	1.04 (0.91–1.18)	0.98 (0.78–1.23)
Antagonist 375 IU	1.11 (0.90–1.37)	1.30 (0.90–1.88)
Antagonist 450 IU	0.94 (0.76–1.17)	0.91 (0.63–1.33)
OPU operator: A	1.00	1.00
B	0.98 (0.91–1.04)	0.90 (0.79–1.01)
C	1.04 (0.94–1.16)	1.03 (0.85–1.24)
D	0.68 (0.51–0.89)	0.79 (0.47–1.37)
E	0.78 (0.71–0.86)	0.85 (0.73–1.00)
F	0.86 (0.78–0.97)	0.77 (0.62–0.97)
G	0.95 (0.87–1.05)	0.91 (0.76–1.09)
H	0.93 (0.84–1.02)	0.94 (0.78–1.12)
I	0.77 (0.70–0.84)	0.83 (0.70–0.98)
J	0.70 (0.56–0.88)	0.56 (0.35–0.91)
Protocol: Old	1.00	1.00
New protocol (V1)	0.87 (0.81–0.93)	0.89 (0.79–1.01)
New protocol (V2 and V3)	0.90 (0.79–1.02)	0.99 (0.79–1.24)
New protocol (V4)	0.84 (0.74–0.94)	0.85 (0.68–1.06)
Patient characteristics		
Attempt No:1st	1.00	1.00
2nd	1.05 (0.99–1.11)	1.03 (0.92–1.15)
3rd or 4th	1.19 (1.07–1.32)	1.08 (0.90–1.29)
Antral follicle count: <10	1.00	1.00
11–16	1.16 (1.11–1.23)	1.14 (1.01–1.27)
16–52	1.29 (1.20–1.38)	1.22 (1.07–1.38)
Age (SDs)	0.87 (0.85–0.89)	0.91 (0.87–0.96)
Age^2 (SDs)	0.96 (0.94–0.99)	0.96 (0.93–1.00)
Log (AMH) (SDs)	1.35 (1.30–1.40)	1.29 (1.21–1.38)
Gonadotropin: HMG	1.00	1.00
rFSH	1.15 (1.07–1.24)	1.13 (0.99–1.29)
Unexplained fertility	1.07 (1.00–1.14)	1.03 (0.91–1.17)
Mild tubal	1.01 (0.94–1.08)	0.96 (0.85–1.10)
Severe tubal	0.92 (0.77–1.09)	0.92 (0.66–1.30)
Mild male factor	0.99 (0.93–1.05)	1.02 (0.92–1.13)
Severe male factor	1.11 (0.88–1.40)	0.96 (0.64–1.44)
Endometriosis	0.94 (0.85–1.06)	0.89 (0.72–1.12)
Endometrioma	0.87 (0.75–1.02)	0.89 (0.68–1.18)
BMI (SDs)	1.01 (0.99–1.04)	1.00 (0.96–1.05)
