# Supplementary material for: How much variation in oocyte yield after controlled ovarian stimulation can be explained? A multilevel modelling study
Source: Hum Reprod Open. 2017 Nov 13;2017(3):hox018. doi: 10.1093/hropen/hox018 (PMC6276674; doi:10.1093/hropen/hox018)
Supplement: Supplementary Data [file hox018suppl_table4.pdf]

Supplementary Table SIV **Yield ratios and 95% CIs from fitted Poisson regression model of number of oocytes with the covariates shown in the table. The estimates do not have a clear interpretation, because total dose and stimulation duration are partially determined by response.**

Parameter	Yield ratio	95% CI	
Intercept	1.81	1.31	2.5
Age (SDs)	0.88	0.85	0.91
Age^2	0.97	0.94	0.99
LDR: 75-150IU	1		
LDR: 187-250IU	1.31	1.15	1.49
LDR: 300IU	1.48	1.27	1.73
LDR: 375IU	1.72	1.29	2.31
LDR: 450IU	1.59	1.21	2.09
Ant:75-150IU	0.80	0.69	0.91
Ant: 187-250IU	1.28	1.05	1.58
Ant: 300IU	1.22	1.05	1.41
Ant: 375IU	1.51	1.19	1.93
Ant:450IU	1.54	1.18	2.02
BMI (SDs)	1.01	1	1.01
log(AMH) (SDs)	1.46	1.37	1.56
Gonadotropin: HMG	1		
rFSH	1.08	1	1.18
AFC: <10			
11–16	1.16	1.09	1.24
16–52	1.29	1.21	1.39
OPU A	1		
OPU B	1.01	0.74	1.38
OPU C	1.29	1.12	1.48
OPU D	1.28	1.13	1.46
OPU E	0.82	0.63	1.08
OPU F	1.31	1.17	1.45
OPU G	1.4	1.22	1.62
OPU H	1.33	1.2	1.47
OPU I	1.16	1.00	1.35
OPU J	1.08	0.95	1.23
Unexplained	1.04	0.97	1.12
mtubal	0.99	0.92	1.07
stubal	0.96	0.8	1.15
mm	0.96	0.9	1.03
sm	1.13	0.88	1.45
endometriosis	0.94	0.84	1.07
endometrioma	0.91	0.78	1.06
Total dose of gonadotropins (SDs)	0.84	0.79	0.89
Stimulation duration (SDs)	1.13	1.09	1.18
Old protocol	1		
New Protocol: V1	0.84	0.78	0.91
V2 & V3	0.85	0.74	0.98
V4	0.81	0.71	0.93
