# Supplementary material for: How much variation in oocyte yield after controlled ovarian stimulation can be explained? A multilevel modelling study
Source: Hum Reprod Open. 2017 Nov 13;2017(3):hox018. doi: 10.1093/hropen/hox018 (PMC6276674; doi:10.1093/hropen/hox018)
Supplement: Supplementary Data [file hox018suppl_figure1.pdf]

Human Reproduction Open, 2017
doi:10.1093/hropen/hox018

human reproduction open

SUPPLEMENTARY DATA

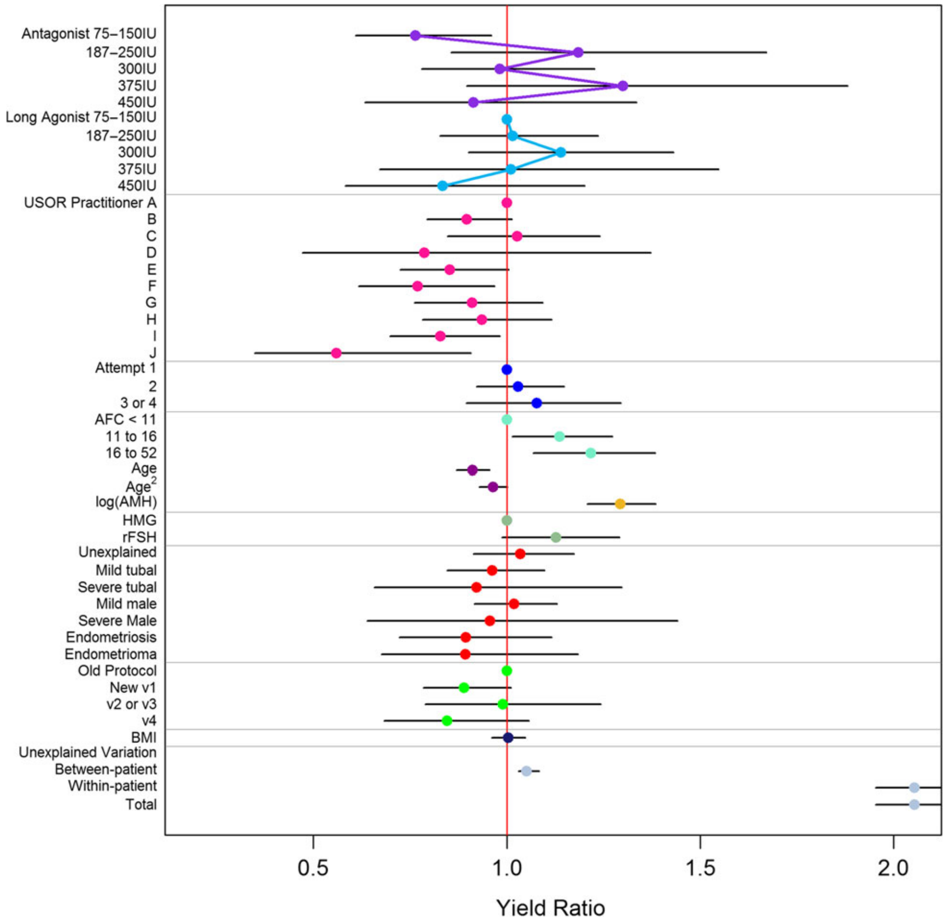

Supplementary Figure S1 Yield ratios and 95% CIs from the multivariable Poisson regression model of number of metaphase II oocytes per cycle. Continuous predictors have been standardized, so that coefficients display the expected multiplicative increase in the yield ratio for a SD change in the variable. Increasing dose effect under a GnRH antagonist regime is shown by the purple connecting line. Increasing dose effect under a GnRH long agonist regime is shown by the blue connecting line.
