# Supplementary material for: How much variation in oocyte yield after controlled ovarian stimulation can be explained? A multilevel modelling study
Source: Hum Reprod Open. 2017 Nov 13;2017(3):hox018. doi: 10.1093/hropen/hox018 (PMC6276674; doi:10.1093/hropen/hox018)
Supplement: Supplementary Data [file hox018suppl_figure2.pdf]

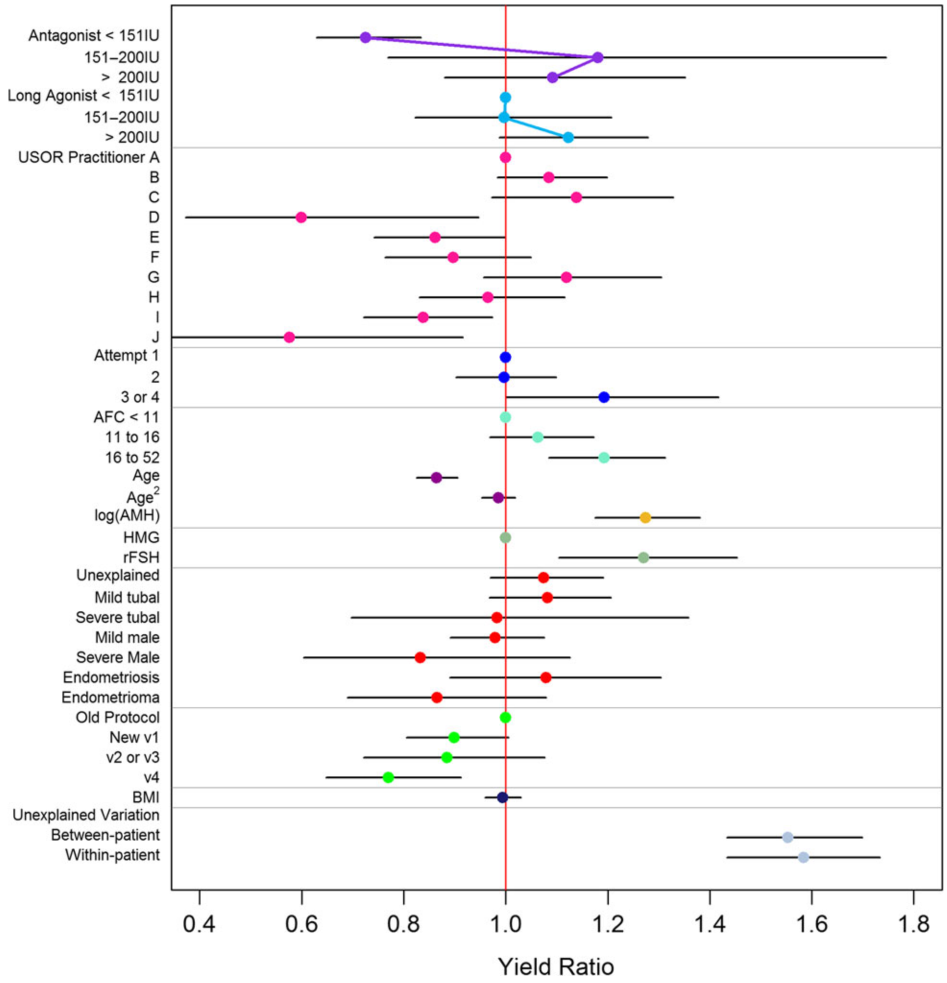

Supplementary Figure S2 Sensitivity analysis: yield ratios and 95% CIs from a multivariable Poisson regression model of number of oocytes per cycle, restricted to low gonadotrophin doses. Continuous predictors have been standardized, so that coefficients display the expected multiplicative increase in the yield ratio for a SD change in the variable. Increasing dose effect under a GnRH antagonist regime is shown by the purple connecting line. Increasing dose effect under a GnRH long agonist regime is shown by the blue connecting line.
